# The Polymorphisms at *PRSS1-PRSS2* and *MORC4* Loci and the Risk of Post-ERCP Pancreatitis

**DOI:** 10.1155/2018/1064783

**Published:** 2018-11-04

**Authors:** Phonthep Angsuwatcharakon, Pimpayao Sodsai, Rungsun Rerknimitr, Nattiya Hirankarn

**Affiliations:** ^1^Department of Anatomy, Faculty of Medicine, Chulalongkorn University, Thai Red Cross Society, Bangkok 10330, Thailand; ^2^Department of Medicine, Faculty of Medicine, Chulalongkorn University, Thai Red Cross Society, Bangkok 10330, Thailand; ^3^Center of Excellence in Immunology and Immune Mediated Diseases, Department of Microbiology, Faculty of Medicine, Chulalongkorn University, Bangkok 10330, Thailand

## Abstract

**Background and Aim:**

The risks of post-ERCP pancreatitis (PEP) are identified as patient- and procedure-related factors. However, the genetic contribution for PEP is still unclear. Recent data show that the polymorphisms of *PRSS1-PRSS2* (*rs10273639*) and *MORC4* (*rs12688220*) are associated with recurrent acute pancreatitis and chronic pancreatitis. We aim to evaluate the association between these polymorphisms and post-ERCP pancreatitis in order to improve better prognosis and better care for these patients.

**Methods:**

This is a retrospective, case-control study which includes 49 cases and 97 controls that are age-, procedure-, and risk of PEP-matched with the cases in 1 : 2 fashion. The PEP was diagnosed and graded for severity according to the standard consensus, and the risk factors of PEP were identified according to the ESGE guideline. Polymorphisms at *rs10273639* and *rs12688220* were evaluated by TaqMan technique and were identified in 133 (40 cases and 93 controls) and 146 patients, respectively.

**Results:**

The demographic data between 2 groups are not significantly different. The genotype frequencies of *PRSS1-PRSS2* (TT, TC, and CC) are 26, 13, and 1 vs. 67, 25, and 1 in cases and controls, respectively (*p* = 0.642). The genotype frequencies of *MORC4* in female (TT, TC, and CC) are 8, 23, and 5 vs. 12, 26, and 21 in cases and controls, respectively (*p* = 0.071). The genotype frequencies of *MORC4* in male (T and C) are 5 and 8 vs. 21 and 17 in cases and controls, respectively (*p* = 0.468). The allelic frequencies of *MORC4* in combination of both genders (T, C) are 44 and 41 vs. 71 and 84 in cases and control, respectively (*p* = 0.431). In PEP cases, the allelic frequencies of *PRSS1-PRSS2* (T and C) are 59 and 13 vs. 6 and 2 in mild and moderate/severe cases, respectively (*p* = 0.633). The allelic frequencies of *MORC4* (T and C) are 38 and 39 vs. 4 and 4 in mild and moderate/severe cases, respectively (*p* = 0.972).

**Conclusion:**

Polymorphisms at *PRSS1-PRSS2* and *MORC4* are not associated with the risk or severity of post-ERCP pancreatitis.

## 1. Introduction

Endoscopic retrograde cholangiopancreatography (ERCP) is a minimally invasive procedure for treatment of pancreaticobiliary diseases. Post-ERCP pancreatitis (PEP) is one of major complications and has a pool incidence rate of 3.5% with a mortality rate of 3% among PEP patients [1]. The risk factors can be divided as patient- and procedure-related factors [1]. The postulated mechanisms of PEP are the obstruction of pancreatic outflow, hydrostatic injury, chemical or allergic injury, infection, enzymatic injury, mechanical pancreatic duct injury, or thermal injury to papilla which induce premature activation of trypsin and subsequent pancreatitis [2]. However, only 2.9–8.6% of patients with definite risk factors develop PEP [1]; therefore, other predisposing factors especially genetic factor should be evaluated for PEP.

Genetic susceptibility was proven to be a predisposing factor in alcoholic pancreatitis by a mechanism of lowering threshold of susceptibility to develop pancreatitis [3, 4]. The polymorphisms at *PRSS1-PRSS2 rs10273739*, which influence trypsinogen expression, and *MORC4 rs12688220*, which associate with atypical localization of cludin-2 in pancreatic acinar cells, are associated with acute pancreatitis [5], related recurrent acute pancreatitis, and chronic pancreatitis [3, 4]. We hypothesized that patients with high susceptibility to pancreatitis might have higher incidence of PEP that those without these genetic risk factors. Therefore, we aimed to investigate the association between the polymorphisms at *PRSS1-PRSS2 rs10273739* and *MORC4 rs12688220* and PEP.

## 2. Materials and Methods

### 2.1. Study Subject

We retrospectively review endoscopy database at the endoscopy unit, King Chulalongkorn Memorial Hospital during 2008–2013. PEP was diagnosed and graded for severity according to the standard consensus [6]. In brief, patient with new onset or worsening of pancreatic-type, epigastric pain and serum amylase at 24-hour post-ERCP of more than 3 times of upper normal limit is diagnosed of having PEP. The severity is classified as mild, moderate, and severe when the length of hospital stay are 2–3 days, 4–10 days, and >10 days, respectively. PEP with hemorrhagic pancreatitis, phlegmon, or pseudocyst or PEP that requires percutaneous drainage or surgery is also classified as severe PEP. The matched controls were identified in 1 : 2 fashion (case : control). The controls were matched with cases according to age, procedure, and risk of PEP. Patients are classified as high-risk when they have at least one of the following factors: history of suspected sphincter of Oddi dysfunction (SOD), precut sphincterotomy, difficult cannulation, more than three pancreatic guidewire passages, and/or injection [1, 7]. Informed consent and blood sample were obtained from cases and controls. The study protocol has been approved by the institutional review board.

### 2.2. DNA Extraction and Genotyping Study

Whole blood was collected with ethylenediaminetetraacetic acid (EDTA) as an anticoagulant. Genomic DNA was isolated from peripheral blood leukocytes using a standard salting out method [8]. The genotyping study of 2 SNPs from 2 genes was performed. Polymorphisms of the PRSS1 gene at rs10273639 (C/T) and MORC4 at rs12688220 (C/T) were identified using the TaqMan SNP genotyping method (ABI Applied Biosystems).

### 2.3. Statistical Analysis

Because *rs12688220* resides at X chromosome, we analyze genotype separately by gender (3 × 2 in female and 2 × 2 in male groups) and analyze allelic frequency of both genders (2 × 2) [9]. Hardy–Weinberg equilibrium (HWE) was determined by Pearson's *χ* ^2^ goodness-of-fit test. The allele and genotype frequencies were performed in the case-control association using the chi-square (*χ* ^2^) test. A *p* value of less than 0.05 was considered statistically significant. Odds ratios (OR) with 95% confidence interval (CI) were calculated by the statistical program Epi Info version 6 [10].

## 3. Results

There were 49 cases and 97 controls included in this study. The mean (SD) age of case and control is 60.3 (14.12) and 60.2 (15.40) years, respectively (*p* = 0.95). The proportion of female gender is 73% and 61% in case and control, respectively (*p* = 0.14). The proportion of high-risk groups is 67% and 64% in case and control, respectively (*p* = 0.63). The risk factors of PEP between 2 groups were not different (Table 1). The severity of pancreatitis was classified as mild (*n* = 45), moderate (*n* = 3), and severe (*n* = 1). The polymorphisms of *MORC4* (*rs12688220*) were identified in all patients, and the polymorphisms of *PRSS1-PRSS2* (*rs10273639*) were identified in 113 patients (40 cases and 93 controls). The genotype frequencies of *PRSS1-PRSS2* (*rs10273639*) (TT, TC, and CC) are 26, 13, and 1 versus 67, 25, and 1 in cases and controls, respectively (*p* = 0.64). The genotype frequencies of *MORC4* (*rs12688220*) in female (TT, TC, CC) are 8, 23, and 5 versus 12, 26, and 21 in cases and controls, respectively (*p* = 0.07). The genotype frequencies of *MORC4* (*rs12688220*) in male (T and C) are 5 and 8 versus 21 and 17 in cases and controls, respectively (*p* = 0.47). The allelic frequencies of *MORC4* in combination of both genders (T, C) are 44 and 41 versus 71 and 84 in cases and control, respectively (*p* = 0.43). In PEP cases, the allelic frequencies of *PRSS1-PRSS2* (T and C) are 59 and 13 versus 6 and 2 in mild and moderate/severe cases, respectively (*p* = 0.63). The allelic frequencies of *MORC4* (T and C) are 38 and 39 versus 4 and 4 in mild and moderate/severe cases, respectively (*p* = 0.97) (Table 2).

## 4. Discussion

This is the first study evaluating the polymorphisms at *rs10273639* and *rs12688220* and the risk of PEP in 49 cases and the 97 matched controls. The controls were matched with cases according to age, procedure, and risk of PEP. The majority of both groups (67% in case and 63% in control) were considered as high-risk. In this study, the genotype and allelic frequencies of polymorphisms at *rs10273639* and *rs12688220* were not significantly different among case and control.

Polymorphisms at *rs10273639* involve in the production of trypsinogen which is an important protein in the development of PEP. The previous study demonstrated that patients with PEP had higher baseline trypsinogen-1 [11] and the serum levels of trypsinogen-1 [11] and -2[12] increased significantly after ERCP when compared with control. Polymorphisms at *rs10273639* influence the regulation of *PRSS1* expression. The *TT* genotype which contributes the lowest expression of trypsinogen is considered as a protective factor for pancreatitis [3]. In our study, there was no association between polymorphism at *rs10273639* and PEP and the proportion of *CC : CT : TT* genotypes was 65% : 32.5% : 2.5% versus 72% : 27% : 1% in case and control, respectively. In our study population, majority of patients in both groups carry *CC* genotypes and *TT* genotype was relatively lower when compared with the study from Europe [4, 5], which had proportions of *TT* genotypes in either acute pancreatitis, alcoholic chronic hepatitis, or control groups ranged from 9 to 18%. The association between polymorphisms at *rs10273639* and acute pancreatitis was not universally identified across European countries in the previous study [5]; the association was found in Germany and Hungary, but not Italy and Poland. Additionally, the study from India [13] demonstrated the proportion of *TT* genotypes in idiopathic recurrent pancreatitis, chronic pancreatitis, and control of 50%, 51%, and 54%, respectively. These demonstrate that the proportion of polymorphisms at *rs10273639* varied among each ethnics and the association was not universal. Hence, the protective effect of *TT* genotype was not demonstrated in our study possibly because of the very low proportion of *TT* genotype in Thai population.

Claudin-2 is normally expressed in tight junction between pancreatic ductal cells [14]. *MORC4* resides in X chromosome and encodes claudin-2 protein [3]. Polymorphism at *MORC4* locus (*rs12688220*) does not alter protein expression [3]. However, *T* (*TT*) genotypes associate with atypical localization of claudin-2 to the basolateral membrane of acinar cells [3] and associate with the development of pancreatitis [3, 4]. Prior studies demonstrated the association between polymorphism at *rs12688220* and all-cause acute pancreatitis, including 56 patients of PEP [5]. However, in subgroup analysis, the associations were presented only in alcoholic acute pancreatitis and patients in Italy and Poland, not Germany and Hungary [5]. In our study, we compared the genotype frequency in male and female and allelic frequency of combined sexes. In female, the proportion of *TT* : *TC* : *CC* was 22% : 64% : 14% versus 20% : 44% : 36% in case and control, respectively. In male, the proportion of *T* : *C* was 38% : 62% versus 55% : 45% in case and control, respectively. There was a trend, but not statistically significant in female gender (*p* = 0.07). In combination of both sexes, the proportion of *T* : *C* was 52 : 48 versus 46 : 54 in case and control, respectively. There was no association between polymorphism at *MORC4* locus (*rs12688220*) and PEP.

Currently, there was one study focused on the association of polymorphism and the risk of PEP. Mystakidis et al. [15] investigated 30 patients with PEP from 2 high-volume endoscopy centers in Greece. The N34S mutation in *SPINK 1* gene was evaluated by allele-specific PCR and RLFP. There was no *SPINK 1* mutation detected in PEP cases; however, the prevalence of N34S mutation in *SPINK 1* gene of the control group was not elucidated in this study. Although we designed a matched case-control study and we were able to identify the proportion of polymorphisms among cases and control, we were not able to identify the association between polymorphisms at *rs10273639* or *rs12688220* with either all PEP or severity subgroup of PEP. Interestingly, we found a trend of significant association between *rs12688220* and PEP in the female subgroup. In order to identify this, a larger sample size is needed to make the conclusion.

## 5. Conclusions

Polymorphisms at *PRSS1-PRSS2* (*rs10273639*) and *MORC4* (*rs12688220*) are not associated with the risk or severity of post-ERCP pancreatitis.

## Figures and Tables

**Table 1:** Patients characteristics and result of *PRSS1-PRSS2* (*rs10273639*) and *MORC4* (*rs12688220*) genotypes.

	Patients with PEP (*n* = 49)	Patient without PEP (*n* = 97)	*p* value
Age, mean (SD) (years)	60.3 (14.12)	60.2 (15.40)	0.954
Gender (female, %)	73%	61%	0.141
Risk group (high risk, %)	67%	64%	0.713
Risk factors			
(i) Sphincter of Oddi dysfunction	1 (2%)	0	0.336
(ii) Precut sphincterotomy	9 (18.4%)	16 (16.5%)	0.777
(iii) Difficult cannulation	13 (25.5%)	19 (19.6%)	0.338
(iv) Pancreatic duct injection or cannulation > 3	11 (22.4%)	12 (12.4%)	0.114
*PRSS1-PRSS2* genotype frequency^∗^ (%)			0.642
TT	26 (65)	67 (72)	
TC	13 (32.5)	25 (26.9)	
CC	1 (2.5)	1 (1.1)	
*MORC4* genotype frequency (%)			
TT (female)/T (male)	8 (22.2)/5 (38.5)	12 (20)/21 (55.3)	0.071 (female)/0.468 (male)
TC (female)	23 (63.9)	26 (44)	
CC (female)/C (male)	5 (13.9)/8 (61.5)	21 (36)/17 (44.7)	
*PRSS1-PRSS2* allelic frequency^∗^ (%)			0.493
T	65 (81.25)	159 (85.5)	RR (95% CI) = 0.81 (0.52–1.28)
C	15 (18.75)	27 (14.5)	
*MORC4* allelic frequency (%)			0.431
T	44 (51.8)	71 (45.8)	RR (95% CI) = 1.17 (0.83–1.66)
C	41 (48.2)	84 (54.2)	

^∗^Results from 133 sample (40 cases and 93 control). PEP: post-ERCP pancreatitis.

**Table 2:** Allelic frequency of *PRSS1-PRSS2* (*rs10273639*) and *MORC4* (*rs12688220*) according to severity grading of PEP.

Allelic frequency	Mild PEP (*n* = 45)	Moderate/severe PEP (*n* = 4)	*p* value
*PRSS1-PRSS2*			0.63
T	59	6	
C	13	2	
*MORC4*			0.97
T	38	4	
C	39	4	

PEP: post-ERCP pancreatitis.

## Data Availability

The genotype data used to support the findings of this study are included within the supplementary information file.

## Supplementary Materials

Supplementary Materials PRSS1-PRSS2 (rs10273639) and MORC4 (rs12688220) genotype data.
